# Case Report: Unprovoked venous thromboembolism in a young adult male

**DOI:** 10.12688/f1000research.18202.2

**Published:** 2019-03-25

**Authors:** Jeyhan Dhabhar, Varshil Mehta, Nimit Desai, Sameer Dawoodi, Sojib Bin Zaman

**Affiliations:** 1 Department of Internal Medicine, MGM Medical College, Navi Mumbai, India; 2 Department of Internal Medicine, Dr. L. H. Hiranandani Hospital, Mumbai, India; 3 Maternal and Child Health Division, International Centre for Diarrhoeal Disease Research, Bangladesh (icddr,b), Dhaka, Bangladesh

**Keywords:** Pulmonary Embolism, Idiopathic, Thromboembolism

## Abstract

A 24-year-old male was presented to us with sudden onset of chest pain and dyspnea for the past one hour. There was no history of calf pain, trauma, surgery, prolonged immobilization, long-haul air travel, bleeding diathesis or any other co-morbidity. The patient denied any addiction history. The heart rate was 114 beats/min, and blood pressure was 106/90 mmHg. Electrocardiogram showed tachycardia with S _1_Q _3_T _3_ pattern. The left arterio-venous Doppler study was suggestive of a thrombus in popliteal vein and sapheno-popliteal junction. The CT-Pulmonary Angiogram scan was suggestive of a massive pulmonary thromboembolism. The patient was thrombolysed with Intravenous Alteplase immediately and was put on tab Rivaroxaban for maintenance. He was later discharged after being stable.

Unprovoked venous thromboembolism (VTE) is very rare and has the potential to lead to pulmonary embolism which could be disastrous, especially in young adults. We present such a case where unprovoked VTE was diagnosed and treated. This case suggests that high clinical suspicion is the key for the diagnosis of acute pulmonary embolism, especially in the absence of history suggestive of deep vein thrombosis.

## Introduction

Venous thromboembolism (VTE) consists of pulmonary embolism (PE) and deep vein thrombosis (DVT). It is one of the leading causes of cardiovascular disability and impaired quality of life. It also causes major long-term complications, which include recurrent VTE, chronic thromboembolic pulmonary hypertension and post-thrombotic syndrome ^1^.

PE is said to be the third leading cause for cardiovascular mortality (after myocardial infarction and stroke). It leads to 100 000 deaths annually globally. It is not only the leading preventable cause of death in admitted patients ^1^ but also the leading cause of deaths in mothers during pregnancy in developed countries ^2^.

Idiopathic or unprovoked VTE is defined as “any VTE in the absence of an identifiable predisposing factor” ^3^. Taking into consideration that unprovoked VTE is very rare, especially in the young adults, we put forward a case report of VTE in a 24-year-old man.

## Case report

A 24-year-old man was brought to the emergency department of a hospital, by his office-colleagues, complaining of sudden onset of chest pain and dyspnea at rest, for the last one hour. It was not associated with sweating, palpitations, cough, hemoptysis, syncope, giddiness, leg pain, pedal edema, fever, rash, or any bleeding manifestations.

History of calf pain, trauma, surgery, prolonged immobilization, long-haul air travel, bleeding diathesis or any other co-morbidity was not reported by the patient. The patient also denied having any addiction history. Family history was found to be insignificant.

On admission, the patient’s heart rate was 114/min, and blood pressure was 106/90 mmHg. His respiratory rate was 22/min, and O _2_ saturation rate was 82% at room air. BMI was 20.76 kg/m ^2^. There was no murmur or gallop on cardiovascular examination. Air entry was reduced in the left infra-axillary region. Also, the detailed examination (including Homan’s and Moses sign) was performed and was deemed unremarkable. Electrocardiogram (ECG) showed tachycardia with S _1_Q _3_T _3_ pattern, and chest X-ray was suggestive of obliteration of left costo-phrenic angle. The D-Dimer (17.31 ug/ml) was elevated, 34 times above the normal upper limit (0.5 ug/ml).

**Figure 1.**
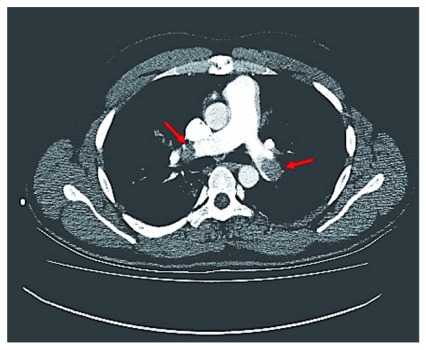
CT Pulmonary Angiogram showing non-lumen occluding thrombus (indicated by red arrows) in the right and left main pulmonary trunk.

CT-Pulmonary Angiogram ( Figure 1) was suggestive of a massive pulmonary thromboembolism. The pulmonary trunk was dilated to ~30 mm. There was a non-lumen occluding circumferential filling defect in the main pulmonary trunk, with maximum thickness of 4.5 mm. A large partial-lumen occluding filling defect was noted in the left main pulmonary artery, which was extending further into the hilar branch, occluding the lumen completely. Another larger complete lumen occluding filling defect was noted in the right main pulmonary artery. These filling defects were extending into the segmental and sub-segmental branches of the lateral segment of the right middle, lingual and bilateral lower lobe. The RV: LV ratio was 2:1. All four pulmonary veins were normal, and there was no evidence of mediastinal pathology. 

On admission, the patient also underwent a bilateral arteriovenous Doppler study, which was suggestive of a partially-lumen- occluding thrombus in the proximal part of left popliteal vein and a completely lumen-occluding thrombus at the left saphenopopliteal junction, approximately 14 cm long. The veins of both legs showed arterialized waveforms.

On 2D echocardiography, right atrium and right ventricle was mildly dilated with grade I, tricuspid regurgitation (TR) and pulmonary arterial systolic pressure by TR jet was 55 mmHg suggestive of moderate pulmonary artery hypertension. No regional wall motion abnormality was observed and left ventricular ejection fraction was 60%.

The coagulation profile was within normal limits. All other blood investigations i.e. hemogram, serum electrolytes, renal and liver function tests were within normal range. The patient had mild hyperuricemia with serum uric acid level being 7.4 mg/dL (Normal: 3.5 – 7.2 mg/dL). Cardiac enzymes (Creatine PhosphoKinase-MB and Troponin T) were mildly elevated. Trop I levels testing was not available at our centre and hence, was not done.

Although it was a clear case of massive VTE, the underlying etiology of such an event could not be extrapolated. Since the patient was a 24-year-old man, without any risk factors or comorbidities, the final diagnosis of unprovoked VTE was made.

The patient was thrombolysed with Injection Alteplase infusion (100mg IV, over two hours) with Injection Enoxaparin 60 mg given subcutaneously every 12 hours. After which, the patient developed hypotension which was treated with inotropic support. Although he had tachycardia post-thrombolysis for the next two days, his blood pressure returned to normal on the third day. 48-hours after giving thrombolytic treatment, the left lower limb venous doppler was done which was suggestive of a partial-lumen occluding the thrombus in popliteal vein extending from saphenopopliteal junction to mid-leg approximately 10 cm long. The CT-pulmonary angiogram was not repeated (post-thrombolysis), due to financial constraints; however, the patient improved drastically. He was shifted to general ward on day 4 post admission. Tab Rivaroxaban 15 mg (12 hourly, orally) was prescribed for the next three weeks. The patient was discharged successfully on the 15 ^th^ day of admission. One week after discharge, the patient was advised Tab. Rivaroxaban 20 mg, once daily. The patient was most satisfied with the treatment and the recovery he’d made, but this was not recorded in a form of any official questionnaire (but he had mentioned this verbally).

The patient had followed up in our center for the first three months, following which relocated to Uttar Pradesh, India (his hometown). He experienced no side effects of Rivaroxaban. At three months of post discharge, a lower limb doppler was repeated which showed more than 50% reduction in size of the obstructing thrombus as compared to the previous one done at admission. Post three months of discharge, the patient has not returned back yet and has been lost to follow up.

## Discussion

VTE in a young male patient is life-threatening, especially if it is unprovoked. The most common and important ECG finding in a patient with PE is sinus tachycardia and the presence of S _1_Q _3_T _3_ pattern in the ECG. Presence of these findings shortened the time for diagnosis of acute PE in our patient and the management after that ^4^.

Literature suggests that the risk of early death among patients with symptomatic PE is 18-fold higher when compared with the patients having DVT alone ^5^. Had this patient presented with sinus tachycardia alone, he would have undergone a battery of investigations, and prompt management (thrombolysis) would have been delayed. Furthermore, the incidence rate of VTE is about 1.5 per 1,000 person-years while appearance of DVT is twice as common ^6^.

Since our patient did not have any clinical features suggestive of DVT, having a Doppler study suggestive of DVT, was more of an additional finding, confirming VTE in this patient. A young man with sudden chest pain and dyspnea without any leg pain and who is otherwise healthy, is likely to be mismanaged, as suspicion of developing pulmonary or venous thrombo-embolism is quite low.

Before attempting to use the Wells criteria, a point-score based on clinical features and the likelihood of diagnoses other than PE, the clinician must first have a suspicion of the diagnosis before attempting to apply the Wells criteria ^7^. On admission, the Wells score for PE was 4.5 with a moderate probability of having a PE for the present patient based on the history and clinical features. The findings on CT Pulmonary Angiogram scan and ECG, increased the possibility of a PE, hence 3 points and 1.5 points were added for presence of tachycardia in our patient’s total score. The well’s score for DVT was 0, as there was no history of cancer, surgery, immobilization, calf swelling, superficial veins, lower limb swelling, tenderness, paralysis or previous history of DVT. Hence high clinical suspicion remains the key in diagnosing PE, especially in the absence of DVT.

A case of unprovoked VTE, especially acute PE, needs to be extensively worked up, to prevent recurrence. Apart from history, family members needs to be screened for coagulopathy or asymptomatic VTE, which was not done in this case. Although, coagulation profile, i.e. International Normalised Ratio (INR), prothrombin time, and activated partial thromboplastin time (APTT), was normal in our patient, it is advisable to do a thrombophilia profile. The thrombophilia profile will help to anticipate the tendency to develop pathological clotting or thrombotic disorders and consists of namely, factor V Leiden mutation, prothrombin gene mutation, protein C resistance, anti-beta 2 glycoprotein, anticardiolipin antibodies, protein S activity and serum homocysteine levels ^8^. Due to the huge cost of these investigations, it becomes difficult to run such investigations while dealing with a case of acute PE in hospital. Thrombophilia profile is not indicated for the patients who are on anticoagulation therapy. Our patient refused to give consent for doing a thrombophilia profile, hence the procedure was not done.

Inj. Alteplase is indicated as a fibrinolytic agent for the lysis of the clot seen in acute massive PE ^9^. Massive PE is caused by obstruction (more than 50% of the cross-sectional area) of the pulmonary arterial tree, leading to an acute and subsequently severe cardiopulmonary failure due to right ventricular overload ^10^.

## Conclusion

Silent VTE can develop into PE which may remain unrecognised for a long time before the clinical features develop. High clinical suspicion is the key, for the diagnosis of acute PE in young patients, especially in the absence of history suggestive of DVT. In the absence of hemodynamic instability, the decision of fibrinolysis, in acute massive PE, largely depends on CT-Pulmonary Angiogram.

## Consent

Written informed consent was taken from the patient and hospital authority to publish this case.

## Data availability

All data underlying the results are available as part of the article and no additional source data are required.
