# Supplementary material for: Case Report: Unprovoked venous thromboembolism in a young adult male
Source: F1000Res. 2019 Mar 25;8:182. Originally published 2019 Feb 14. [Version 2] doi: 10.12688/f1000research.18202.2 (PMC6446494; doi:10.12688/f1000research.18202.2)
Supplement: Peer review history

# Peer review: Reviewer response for version 2

**DOI:** 10.5256/f1000research.20440.r46157

**Published:** 2019-04-01

**Authors:** Shakti A. Goel

**Affiliations:** 1 Department of Spine Surgery, Indian Spinal Injuries Center, New Delhi, Delhi, India

I believe that the authors have answered the queries meticulously and the article may be considered for indexing now.

I have read this submission. I believe that I have an appropriate level of expertise to confirm that it is of an acceptable scientific standard.

# Peer review: Reviewer response for version 1

**DOI:** 10.5256/f1000research.19910.r45517

**Published:** 2019-03-18

**Authors:** Himel Mondal

**Affiliations:** 1 Department of Physiology, Fakir Mohan Medical College and Hospital, Balasore, Odisha, India

**Overall evaluation**

 This is an interesting case report of venous thromboembolism in a young adult male without any predisposing factors. The authors described the case in detail with relevant and recent literature references. However, there is a need of a minor revision to enrich the manuscript. The author may revise the manuscript keeping in following points in mind:

**Title**

 As this is a case of a “young adult male,” authors may add the word “male” in the title of the manuscript.

**Abstract**

 The patient presented with chest pain and dyspnoea. The second line of the “Abstract” described that the patient did not have any “calf pain.” Authors could add some cardiological history. They could also add a glimpse of clinical examination (especially heart rate and rhythm) rather than starting with “ECG showed tachycardia…”

**Keywords**

 Authors may add more keywords according to MeSH for wider dissemination of the article.

**Introduction**

 PE causes “100000 deaths” annually. Is it global or regional data? Please specify.

**Case report**

 Authors may add the time when the patient was brought to the hospital, along with the nature of his works in previous 12 hours, if the information is available.

 What was the rhythm of the pulse in clinical examination? If the data is available, the authors may add it.

**Discussion**

 Discussion is adequate and well written.

**Conclusion**

 Written well and it is within the scope of the article.

**References**

 References are relevant and recent.

I have read this submission. I believe that I have an appropriate level of expertise to confirm that it is of an acceptable scientific standard.

# Response

**Published:** 2019-03-18

**Authors:** Sojib Bin Zaman

**Affiliations:** International Centre For Diarrhoeal Disease Research, Bangladesh (icddr,b), Bangladesh

Thank you for your reviews and approving it.

**Changes we made:**

**In title:** We have added "male" in the title.

**Abstract**: We have also added heart rate and bp in the abstract.

**Introduction:** It leads to 100 000 deaths annually globally

 The heart rhythm was normal and hence we didn't add it.

 Hopefully, we have made adequate changes. Once again thank you for reviewing for us.

 Sojib.

# Peer review: Reviewer response for version 1

**DOI:** 10.5256/f1000research.19910.r44458

**Published:** 2019-02-15

**Authors:** Shakti A. Goel

**Affiliations:** 1 Department of Spine Surgery, Indian Spinal Injuries Center, New Delhi, Delhi, India

This is an interesting case report which discusses a rare phenomenon of unprovoked venous thromboembolism and its management. I would congratulate the authors for their work. However, there are a few concerns:

 Though the authors have mentioned that there was no history of long air travel, it will be helpful to know if the patient had any history of long car travel before the event precipitated. It has been noted that DVT is commonly encountered in patients who undergo long car travel. Such information, if added, shall make the physicians more aware.

 Did the physicians/authors conduct any clinical examination to test DVT. Were Homan's or Mosses sign performed. If yes, what were the results. If not, it will be helpful to know the reason for not performing them. A little detail on clinical examination of the patient should also be added to the manuscript besides the symptoms and findings.

 It will also be helpful to know the Trop I values, if they were assessed by the authors.

 The authors have mentioned that patient was kept on Rivaroxaban. Were there any side effects that were encountered. It will also be helpful if the authors could mention about the follow-up period of the patient in the manuscript.

 Was any assessment done in the post discharge period. Was there any change in vessel size? Any adaptive remodeling seen with the treatment using Rivaroxaban .

 Was the patient satisfied with the treatment? Any satisfaction index which was assessed. Authors may add it if found to be appropriate. They could use the attached reference for the same.

I have read this submission. I believe that I have an appropriate level of expertise to confirm that it is of an acceptable scientific standard, however I have significant reservations, as outlined above.

## References

[1] GuoLWWangBGoelSALittleCTakayamaTShiXDRoenneburgDDiRenzoDKentKC: Halofuginone stimulates adaptive remodeling and preserves re-endothelialization in balloon-injured rat carotid arteries. *Circ Cardiovasc Interv*.2014;7(4) : 10.1161/CIRCINTERVENTIONS.113.001181 594-601 10.1161/CIRCINTERVENTIONS.113.001181 25074254PMC4140988

[2] SrivastavaNGoelS: A study of patient satisfaction level in inpatient spine department of a tertiary care multi-speciality hospital. *Journal of Medical Research and Innovation*.2018; 10.15419/jmri.147 10.15419/jmri.147

[3] GoelSAGuoLWLiuBKentKC: Mechanisms of post-intervention arterial remodelling. *Cardiovasc Res*.2012;96(3) : 10.1093/cvr/cvs276 363-71 10.1093/cvr/cvs276 22918976PMC3500049

# Response

**Published:** 2019-02-15

**Authors:** Sojib Bin Zaman

**Affiliations:** International Centre For Diarrhoeal Disease Research, Bangladesh (icddr,b), Bangladesh

Thank you Dr for reviewing our paper and taking your time out for the same.

Please find my replies to your queries.

- There was no history of prolonged air or car travel. Also, the detailed examination was performed and was deemed unremarkable; hence was not mentioned here (including Homan's and Moses sign).
- Trop I level test are not available in our center, hence was not done, However, we did Trop T and CPK MB, which were normal.
- The patient had followed up in our center for the first three months, following which relocated to Uttar Pradesh, India (his hometown). He experienced no side effects of Rivaroxaban. At three months, post discharge, a lower limb doppler was repeated which showed more than 50% reduction in size of the obstructing thrombus as compared to the previous one done at admission. Post three months of discharge, the patient has not returned back yet and has been lost to follow up.
- The patient was most satisfied with the treatment and the recovery he'd made, but this was not recorded in a form of any official questionnaire (but he had mentioned this verbally); hence not mentioned here.

Hopefully, these comments will satisfy the queries. If you have any more queries, please feel free to post and we shall be happy to reply them.

# Response

**Published:** 2019-02-19

**Authors:** Shakti A. Goel

**Affiliations:** Indian Spinal Injuries Centre, India

I had an opportunity to go through the review/reply and believe that the authors have addressed the comments meticulously. The article can be accepted once these details have been added to the article.

 This article will certainly be very helpful for the physicians around the globe especially in low to middle income countries.
